# Interprofessional Education and Medical Libraries: Partnering for Success

**DOI:** 10.5195/jmla.2018.464

**Published:** 2018-07-01

**Authors:** Eleanor Shanklin Truex

**Affiliations:** Medical Librarian, Lakeshore Region, Presence Saint Joseph Hospital, Chicago, IL, and Presence Saint Francis Hospital, Evanston, IL

The need for synthesis and collaboration across health care professions is vital to safe and efficient health care delivery. Edwards’s practical treatment of the educational development needed to obtain this goal is a welcome addition to that end. Delineated in nine chapters, this book covers everything from “soup to nuts,” starting with the history of interprofessional education (IPE) to actual case scenarios of program development, with two full chapters devoted to medical/health sciences librarians/libraries and IPE.

As stated in the preface:

The purposes of this book are to describe the variety of Interprofessional education programs in both didactic and clinical settings, discuss how libraries are partnering to further the success of these programs, and expand the notion of “Interprofessional” beyond the typical health professions. It was designed with a variety of audiences in mind: medical educators new to Interprofessional education, experienced IPE practitioners, and medical librarians who want to learn more about IPE and the ways in which libraries can support Interprofessional initiatives on their campus. (p. xiii)

Drawing from a variety of contributors across a widespread geography, this book does all that and more. Each chapter encapsulates an aspect of IPE theory and application. It can be read in its entirety for a solid overview of the history of IPE, including theories regarding IPE and clinical applications of IPE, or the reader can simply read pertinent chapters without digesting the entire tome. This is a benefit to busy clinicians and educators.

The book’s content benefits from many graphs, images, and tables that clearly lay out what can be a dry and abstract subject. The tone is somewhat uneven from chapter to chapter (most likely due to each chapter having been written by a different contributor), but not enough to be jarring to the reader. The more clinically focused chapters tend toward the conversational in tone. For example, one chapter provides a case study that breaks down the authors’ IPE development with a list of concrete, straightforward tips, an approach that most busy educators will welcome. One of the more important features is the final chapter, “Assessing Interprofessional Education.” This is a boon for the educator who inherits an existent IPE program and provides the founders of such a program a tool for evaluating their programs and their evolution. The book includes an index of all contributors with short professional biographies of each; each chapter has a list of references as well.

Drinka and Clarke’s *Healthcare Teamwork: Interprofessional Practice and Education* (Praeger, 2016) is a book with a similar take on this topic, but it appears to be more clinically focused. It contains a chapter, “Patient as Teacher and Learner,” that, as a subject, this book lacks. It also features an appendix of resources for program developers that would be a welcome addition to this book.

This book is of practical use as a reference book for universities or health care systems looking to synthesize their approaches to practice and, ultimately, improve patient care. Medical librarians, given that they touch all departments in any health system, are a natural fit for IPE program involvement. One institution wrote that its library was chosen to develop its IPE *because* it was perceived as neutral in the institution—a telling word (and absolutely true), given the silo-ing that occurs in preclinical education. Edwards’s book would prove useful to a variety of health care professionals: librarians and all clinical educators or program directors, even specialty area administrators looking to foster communication between specialties or to strengthen all practice by supporting collaboration.

